# Anti-HIV Double Variable Domain Immunoglobulins Binding Both gp41 and gp120 for Targeted Delivery of Immunoconjugates

**DOI:** 10.1371/journal.pone.0046778

**Published:** 2012-10-04

**Authors:** Ryan B. Craig, Christopher M. Summa, Miriam Corti, Seth H. Pincus

**Affiliations:** 1 Department of Microbiology, Immunology, and Parasitology, LSU Health Sciences Center, New Orleans, Louisiana, United States of America; 2 Research Institute for Children, Children’s Hospital, New Orleans, Louisiana, United States of America; 3 Department of Computer Sciences, University of New Orleans, New Orleans, Louisiana, United States of America; 4 Department of Pediatrics, LSU Health Sciences Center, New Orleans, Louisiana, United States of America; Tulane University, United States of America

## Abstract

**Background:**

Anti-HIV immunoconjugates targeted to the HIV envelope protein may be used to eradicate the latent reservoir of HIV infection using activate-and-purge protocols. Previous studies have identified the two target epitopes most effective for the delivery of cytotoxic immunoconjugates the CD4-binding site of gp120, and the hairpin loop of gp41. Here we construct and test tetravalent double variable domain immunoglobulin molecules (DVD-Igs) that bind to both epitopes.

**Methods:**

Synthetic genes that encode DVD-Igs utilizing V-domains derived from human anti-gp120 and anti-gp41 Abs were designed and expressed in 293F cells. A series of constructs tested different inter-V-linker domains and orientations of the two V domains. Antibodies were tested for binding to recombinant Ag and native Env expressed on infected cells, for neutralization of infectious HIV, and for their ability to deliver cytotoxic immunoconjugates to infected cells.

**Findings:**

The outer V-domain was the major determinant of binding and functional activity of the DVD-Ig. Function of the inner V-domain and bifunctional binding required at least 15 AA in the inter-V-domain linker. A molecular model showing the spatial orientation of the two epitopes is consistent with this observation. Linkers that incorporated helical domains (A[EAAAK]_n_A) resulted in more effective DVD-Igs than those based solely on flexible domains ([GGGGS]_n_). In general, the DVD-Igs outperformed the less effective parental antibody and equaled the activity of the more effective. The ability of the DVD-Igs to deliver cytotoxic immunoconjugates in the absence of soluble CD4 was improved over that of either parent.

**Conclusions:**

DVD-Igs can be designed that bind to both gp120 and gp41 on the HIV envelope. DVD-Igs are effective in delivering cytotoxic immunoconjugates. The optimal design of these DVD-Igs, in which both domains are fully functional, has not yet been achieved.

## Introduction

Antibodies to the HIV envelope protein (Env, consisting of the precursor gp160, external domain gp120, and transmembrane domain gp41) provide the neutralizing components necessary for an effective AIDS vaccine [1]–[3]. Passive administration of anti-Env antibodies (Abs) may be used as post-exposure prophylaxis, to prevent vertical transmission of HIV infection, or as an adjunct to conventional antiviral therapy [4]–[9]. Our laboratory has been using anti-Env Abs to target cytotoxic anti-HIV immunoconjugates (ICs) as a method to eliminate the persistent reservoir of latently-infected cells and eradicate HIV infection [10]–[15]. Such ICs would serve as the purge agent in so called “activate-and-purge” protocols [16]–[22]. Env is the only HIV protein displayed fully intact on the surface of HIV-infected cells, and there are two well-defined regions of Env that are highly effective targets for delivery of cytotoxic conjugates. They are: 1) the CD4-binding site of gp120, targeted with either CD4-itself or Ab [21], [23]–[29], and 2) the hairpin loop of the membrane distal immunodominant region of gp41, a region that interacts with gp120 [13]–[15], [30]. *In vivo* antiviral activity of these ICs has been demonstrated in mice [15], [25] and macaques (S.H. Pincus, unpublished), and we are continually screening the IC activity of new anti-Env Abs as they are described (references [12]–[15] and S.H. Pincus, unpublished). In this manuscript we propose a novel approach for developing anti-Env Abs to target and kill HIV-infected cells.

Dual variable domain immunoglobulins (DVD-Igs) are immunoglobulin-derived molecules that contain two unique variable domains (V domains) linked to a constant region with the capability of tetravalent, bispecific binding, while retaining affinity and specificity of each of the parental Abs [31]–[34]. For example, DVD-Igs have been constructed that can bind both IL1α and IL1β, or IL-12 and IL-18 [34]. Each of these DVD-Igs has been proven effective in vitro and in vivo, and retains pharmacokinetic properties of the parental Abs [31], [34]. The idea of targeting two separate antigenic sites with a single Ab has also been directed against HIV. The most common approach has been to construct dual domain Abs using an anti-gp120 V-region fused to CD4 [35]–[38]. When the inter-domain linker length was optimized, enhanced neutralization by these CD4-anti-gp120 immunoadhesins was obtained. Mouquet *et al*. made bispecific Abs with one V-domain against gp41 and one against gp120 [39]. Athough the gp41 parental Ab did not neutralize, the bifunctional Ab had enhanced neutralizing ability.

In the studies described here, we seek to design DVD-Igs that can most effectively deliver cytotoxic ICs to cells expressing HIV Env on their cell surface. To this end, we have chosen as the V-region donors the two Abs that have been shown to be most effective at delivering ICs: HY, an affinity matured version of the CD4 binding site Ab b12 [16], [21], [23], and 7B2, which binds to the gp41 loop region [13]–[15]. We have chosen to make DVD-Igs, rather than a bifunctional Ab as did Mouquet, et al, because the DVD-Igs are potentially tetravalent. Native HIV Env exists as trimers of gp120/gp41, and so a multivalent Ab may produce higher avidity interactions. While others have sought Abs with greater HIV-neutralizing activity, the goal of our studies is to produce better anti-HIV ICs. Here we systematically examine the role of linker length, linker type, and domain orientation on binding and effector functions of the DVD-Igs. The results show that the design of the V-domains can determine which biological functions will predominate. Results show the DVD-Igs always outperform the less effective parental Ab and generally equal the activity of the better one, in some cases exceeding the function of the better.

## Materials and Methods

### Reagents and Cells

HEK-derived Suspension 293F cells (Invitrogen, Carlsbad, CA) were maintained in serum-free Freestyle expression medium (Invitrogen), shaking at 120 rpm in 8% CO_2_ at 37° for transient transfection. H9 cells, a human CD4+ lymphoma cell line, were obtained from Dr. M. Reitz (Institute of Human Virology, Baltimore, MD) [40]. H9/NL4-3 cells are persistently infected with the NL4-3 molecular clone of HIV [41] and retain a productive infection in virtually 100% of tissue culture cells [15], [42]. TZM-bl cells (AIDS Research and Reference Reagent Program, ARRRP) are HeLa expressing CD4, CCR5, and CXCR4, with HIV-tat inducible luciferase and beta-galactosidase reporter genes [43]–[45]. H9/NL4-3 and TZM-bl cells were maintained at 37° in 5% CO_2_ in RPMI 1640 medium with 10% fetal bovine serum (Gibco Invitrogen, Grand Island NY) as described elsewhere [12].

HIV isolates used in these studies include NL4-3 (X4-tropic), Ba-L (R5-tropic), 92HT594 (X4/R5), 92HT599 (X4), and MN (X4) [41], [46]-[49]. All isolates were obtained from ARRRP and grown in PHA blasts, with the exception of NL4-3 which was produced by the H9/NL4-3 cell line.

Soluble CD4 (sCD4; ARRRP [50]) was used to observe CD4-mediated effects. Goat anti-human IgG (heavy + light chains) Ab was conjugated to either alkaline phosphatase (AP) or fluorescein isothiocyanate (Invitrogen).

### Design and Production of Antibodies

Parental antibody sequences derive from IgG1/kappa human antibodies HY (Genbank accession numbers JX188440 and JX188441), an affinity matured version of the anti-CD4 binding site Ab b12 [23], and 7B2 (Genbank accession numbers JX188438 and JX188439) which binds gp41 at the site of interaction with gp120 at AA 598–604 (CSGKLIC) [15]. Two additional mutations (T250Q and M428L) were introduced into the constant region of the heavy chain to increase *in vivo* half-life of antibody [51]. DVD-Ig protein sequences were designed *in silico* and DNA synthesized de novo (GenScript, Piscataway, NJ). DNA sequences were codon-optimized and cloned into the eukaryotic expression plasmid pcDNA3.1 (Invitrogen) using either restriction enzyme sites XbaI and PmeI for the heavy chain, or HindIII and EcoRI for the light chain.

Heavy and light chain plasmids were incubated with 293Fectin, a cationic lipid-based reagent, then transfected into suspension 293F cells at an equimolar ratio using the 293Fectin Transfection System (Invitrogen). Supernatant was collected on days 3 and 7 and purified by affinity chromatography on Protein A agarose beads (Invitrogen), eluted with acidic glycine (pH 2.8), neutralized with 2M Tris, concentrated to ∼200 µl using a Microcon YM-30 k centrifugal filter (Millipore, Billerica, MA), and dialyzed in 1x PBS. All antibody concentrations were measured by bicinchoninic acid protein assay (Pierce, Rockford, IL) and confirmed using OD_280_ reading by Nanovue UV Spectrophotometer (GE Healthcare, Piscataway, NJ). We used microcapillary electrophoresis (Agilent Bioanalyzer, GE Healthcare) to determine molecular size and purity of products.

**Figure 1:**
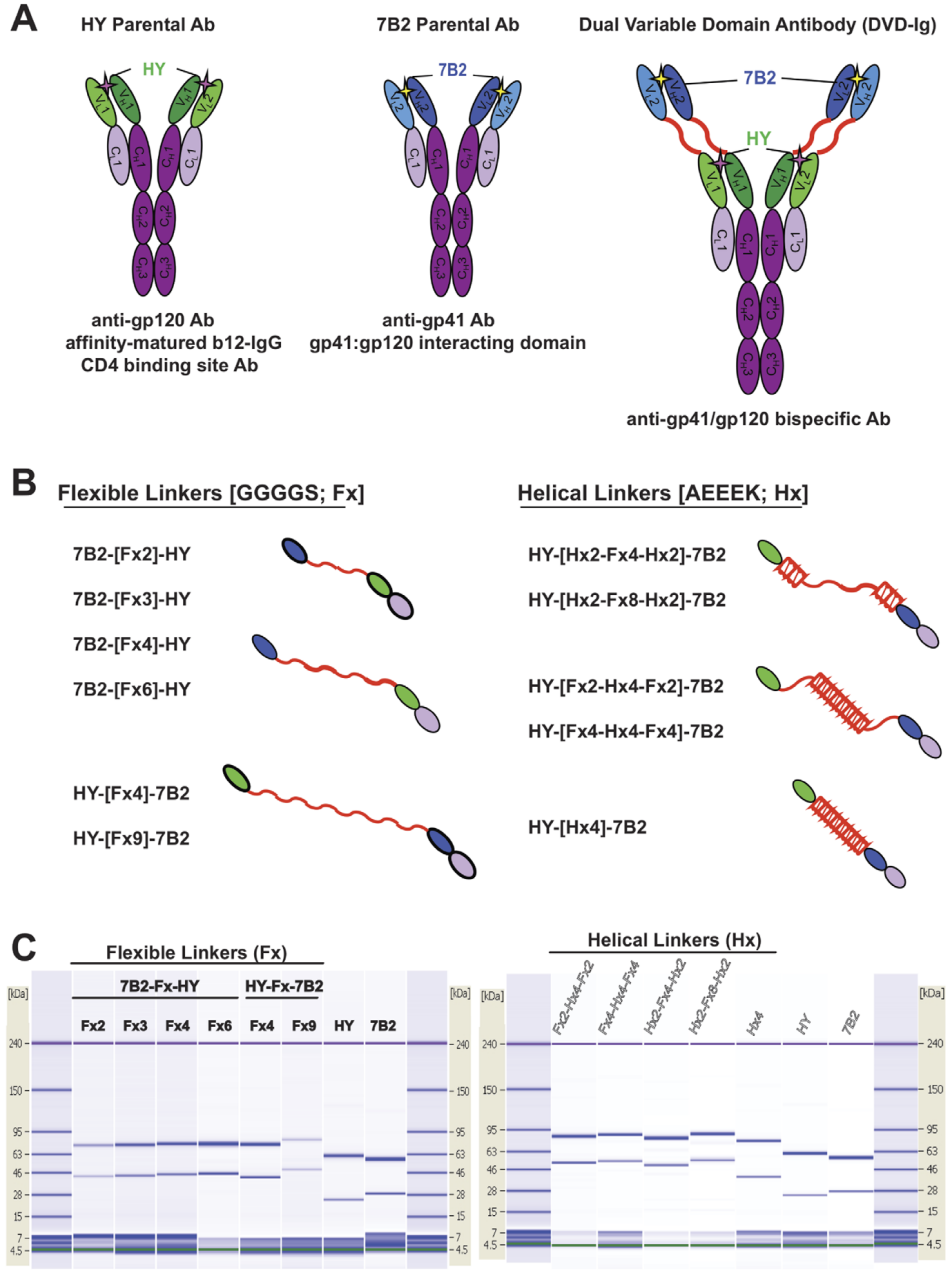
Design and analysis of DVD-Igs. *A.* The overall structures of the two parent antibodies and the DVD-Igs are shown. In this case the DVD-Ig has the 7B2-derived V domains in the external or outer position, HY domains are inner. Throughout the manuscript, the HY antibody is shown in green, 7B2 in blue. *B.* General design of inter-V-domain linkers, and nomenclature used to identify the DVD-Igs having different linkers. Green indicates the HY V domain, blue the 7B2 V domain, the linker is red, and in violet is the first domain of the C region. *C.* Microcapillary electrophoresis of reduced DVD-Igs and parental mAbs. Results are displayed in the familiar format of a coumassie stained gels. Size standards are indicated on the side of each “gel”.

7B2 and HY variable domains were initially joined by “flexible linkers” (Fx) of the repeating sequence: [GGGGS]_n_, where n = linker repeats of 2, 3, 4, 6, or 9, or with no linker at all. The two V-domains were on human IgG1 and κ constant regions, creating a full length DVD-Ig. Constructs were engineered to express each V-domain as either “outside” (distal) or “inside” (proximal). Subsequent constructs with HY as the outer domain incorporated “helical linkers” with 4 repeats of the rigid sequence: A[EAAAK]_4_A (Hx) alone or in conjunction with flexible repeats [52], [53]. Figure 1 shows the DVD-Ig constructs in a schematic manner. The colors and symbols used to identify the constructs are established in figure 1, and maintained throughout all figures in the manuscript.

### ELISA

Antibodies were characterized based on binding to each cognate antigen in an indirect ELISA [12]. Immulon 2HB plates (Thermo, Waltham, MA) were coated with 1.0 µg/ml of antigen – gp41, gp120, gp140, or gp160– in 100 µl PBS per well and incubated at 4° overnight. The gp41 antigen is a linear peptide sequence [LGIWGCSGKLICTT] representing the epitope of 7B2. Gp120, gp140, and gp160 antigens are all recombinant proteins expressed in mammalian cells. Recombinant gp120 antigens represent HIV isolates: IIIB and MN (gifts from Genentech, S. San Francisco, CA), SF162 (ARRRP), and Ba-L (ARRRP). YU-2 (AP97 YU2gp140 Foldon, a gift of R.Wyatt, NIH) is a trimeric version of gp140 that was used to test binding to multimers. Recombinant gp160s (Quality Biological, Gaithersburg, MD) were IIIB or MN/LAI, the latter containing the gp120 portion of MN and the gp41 portion of LAI. Plates were washed with ELISA wash buffer (0.1% tween 20 (Fisher) in PBS) and blocked with PBS/BSA/0.01% sodium azide (PBA) 200 µl per well for 8–16 hr at 4°. After blocking, 100 µl of each test antibody was added in serial dilutions, and incubated at 4° overnight. Next, the plates were washed 6X, then AP-conjugated goat anti-human IgG (H+L chain specific) secondary antibody was added at 1 µg/ml in PBA and incubated for 4 hr at room temperature. AP substrate (4-nitrophenyl phosphate, Sigma) was dissolved in diethanoleamine (buffer to a final concentration of 9.8% (pH = 9.8). After the final wash, 150 µl of substrate was added to each well. ELISA plates were read at 405 nm at room temperature in a BioTek EL320 microplate reader (BioTek, Winooski, VT) at 5–15 min intervals. Time points shown in figures have been chosen so that maximal binding was less than 1.5 to 2.0, within the dynamic range of the reader. Data are presented as the mean and the SEM of triplicate samples.

### Indirect Immunofluorescence and Flow Cytometry

H9/NL4-3 cells (1×10^5^) were stained for flow cytometry in 100 µl in round-bottom 96 well plates (Costar, Lowell, MA) [13]. Serial dilutions of antibody in PBA were added to the cells in the presence or absence of 500 ng/ml of sCD4. Cells were incubated 1 hr at room temperature, washed, and then stained with FITC-conjugated goat anti-human IgG (H+L chain specific) secondary antibody for 1–4 hr, washed twice and fixed in 100 µl of 2% paraformaldehyde. After a minimum of 4 hr, 150 µl of PBS was added. Cells were analyzed on a Becton-Dickson LSR II (BD, Franklin Lakes, NJ) with HTS plate reader. 10000 events were collected and data analyzed by Flow-Jo software (Treestar, Ashland, OR). Forward scatter (FSC) and side scatter (SSC) gated data are represented as either overlayed histograms or as graphs of median fluorescence. None of the parental or DVD-Igs bound to uninfected H9 cells (not shown).

### Surface Plasmon Resonance

The kinetics of Ab binding to immobilized gp160 were determined by surface plasmon resonance using Biacore 3000 (Piscataway, NJ). Recombinant gp160 was immobilized onto CM5 sensor chips by amine coupling at 25 µg/ml (∼1000 RU) in 10 mM sodium acetate pH 5.5. Decreasing concentrations of Ab (400 nM to 0.39 nM in 2X dilutions) in 75 µl PBS with 0.025% P20 detergent were passed over the sensor chips for 150 sec at 30 µl/min (association), followed by a dissociation phase of 20–60 min. Studies were performed at 37°. Chips were regenerated and Ab removed with 15% acetonitrile in 50 mM NaOH. Affinity of each Ab was measured in 3–6 separate experiments, and curves were fit to a bivalent analyte binding model with global Rmax.

### Cytotoxicity of ICs

An indirect cytotoxicity assay was performed to screen unconjugated antibodies for their ability to kill infected cells [54], [55]. H9/NL4-3 cells (8×10^3^) were plated in triplicate in cRPMI in 96 well flat-bottom tissue culture plates (Costar). Controls included: no cells (background) and cells in the absence of antibody/IC (uninhibited). Serial dilutions of antibodies were incubated with cells for 1 hr in the presence or absence of 300 ng/ml of sCD4 in RPMI. The secondary IC was affinity purified goat anti-human IgG (Invitrogen) conjugated to deglycoslyated ricin A chain by the long chain heterobifunctional cross linking reagent succinimidyl 6-[3(2-pyridyldithio) propionamido] hexanoate (Pierce), using protocols described elsewhere [10], [12], [14]. The secondary IC was added to a final concentration of 500 ng/ml. The plates were then incubated for 3 days. For the final 6 hr of incubation, MTS/PMS substrate (Promega, Madison, WI) was added to each well and plates read hourly at 490 nm. Results represent the mean and SEM of triplicate samples, and are plotted as A_490_ with the no cell background subtracted. Under these conditions, there was no cytotoxicity on uninfected H9 cells (not shown). To determine whether the IC activity of the DVD-Ig represented an improvement over that of the parental Abs, we performed a one-tailed t-test comparing the DVD-Ig to the most effective parent mAb at each concentration.

### Neutralization Assay

TZM-bl cells were used in a luciferase-based virus neutralization assay [43], [44], [56]. Each antibody was assayed in triplicate. Experiments included: background controls (cells, no virus, no antibody) and infected cells in the presence or absence of antibody. TZM-bl cells (4×10^4^ cells/ml) were plated in 96-well plates with black sides and clear, flat bottom wells (Costar) and incubated overnight at 37° to allow attachment. The following day, 50 µl of serially diluted antibodies in RPMI were mixed with 50 µl of a pretitered concentration of virus and incubated for 1 hr at room temperature and added to the cells in the presence of diethylaminoethyl dextran (Sigma) 15µg/ml, and incubated for 6 hr at 37°. Medium was added for a total volume of 200 µl/well and plates incubated 48 hr. For luciferase assays, medium was aspirated and 50 µl Bright-Glo Lysis buffer (Promega) was added. Samples were frozen and thawed once, and incubated for 6 hr at room temperature with orbital shaking. Then 10 µl of Bright-Glo luciferase substrate (Promega) was added and luminescence read on Bio-Tek KC4 plate reader as relative luminescence units. Results are displayed as percent neutralization (virus/no Ab = 0%; no virus = 100% neutralization) according to the formula: [1-(RLU_Ab_-RLU_bkgrd_)/(RLU_NoAb_-RLU_bkgrd_)]*100.

## Results

### Production and Characterization of DVD-Ig

Engineered DVD-Ig sequences of heavy (H) and light (L) chains from parental antibodies, including linkers, were synthesized *de novo* on separate plasmids. More specifically, for each chain a variable domain from one parental antibody (eg. 7B2) was fused at its C terminus to a linker, then to the other parent’s (HY) full-length immunoglobulin gene (variable and constant domains). The two plasmids encoding H and L chains were cotransfected into 293F cells to create a bispecific, tetravalent IgG1 antibody with dual variable domains (figure 1A). Inter V-domain linkers were designed based on spatial and functional requirements (figure 1B) [33], [52], [57]–[59]. Abs were produced in cell supernatants and purified over Protein A agarose beads. Abs were tested for purity and concentration before beginning any binding or functional assays. Purity and size of all Abs was examined by microcapillary electrophoresis (figure 1C). Results demonstrate that full length DVD-Igs can be engineered and produced with dual V-domains with various linker types and lengths, resulting in H and L chains of the appropriate size.

### Binding of DVD-Igs to Recombinant and Native Antigen

**Figure 2:**
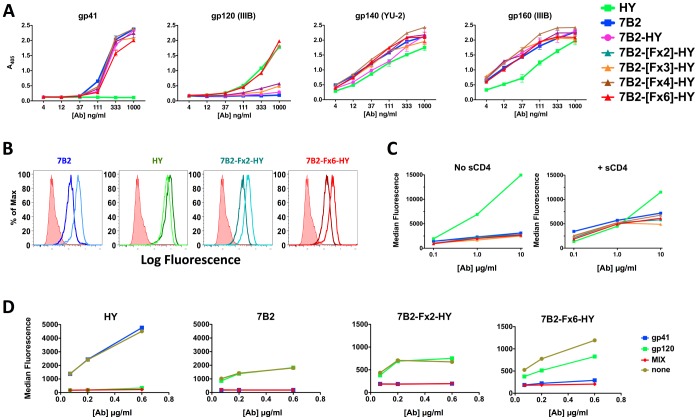
Effect of linker length on the binding of 7B2-Fx-HY DVD-Igs to Env. ELISA (panel A) and flow cytometry (B-D) were used to evaluate the binding of DVD-Igs to Env. *A,* Various forms of HIV Env were adhered to ELISA plates. The gp41 Ag was a peptide representing 7B2’s epitope; gp120 and gp160 were monomeric recombinant proteins; gp140 was a trimeric recombinant protein. Ab binding was detected with AP-conjugated secondary Ab. Results show mean and standard error of duplicates. Where no error bars are visible they are obscured by the symbol. Results are representative of at least 3 different assays (varying by Ab, or Ag tested). *B,* Flow cytometry histograms using secondary immunofluorescence to detect binding of Abs to persistently-infected H9/NL4-3 cells. Abs were tested at 10 µg/ml in the presence (lighter lines) or absence (darker lines) of sCD4 at 300 ng/ml. Isotype control is shown as red shaded histogram. Results are representative of at least 3 different assays (varying by Ab). *C,* Ab binding to H9/NL4-3 cells was detected as in panel *B* but plotted as median fluorescence versus Ab concentration. *D,* Flow cytometry was used to study inhibition of Ab binding to H9/NL4-3 cells by soluble gp41 and gp120 Ags, at 10 µg/ml. Ags indicated by legend. Results are representative of 2 different assays.

First, we sought to determine the effect of linker length on the binding abilities of the inner and outer domains of the DVD-Igs. To do this, a series of constructs were created all with the HY domain on the inside and the 7B2 domain on the outside (figures 1A and 1B). They differed only in the number of repeats of the flexible linker sequence – [GGGGS]. To examine the ability of these DVD-Igs to bind their Ags, ELISA plates were coated with peptide or recombinant antigen, incubated with serial dilutions of DVD-Igs or parental Abs, and probed with an AP-conjugated anti-IgG secondary Ab (figure 2A). All of the DVD-Igs bound the gp41 peptide equivalently to the parental anti-gp41 Ab (7B2), although the DVD-Ig with six flexible linkers (F×6) may have bound slightly less well. It was apparent that the activity of the inner HY domain was hindered in constructs with 3 or less repeats, but 4 and 6 repeats increased binding to gp120 so that it equaled the binding of the anti-gp120 mAb HY. The longer DVD-Igs bound better than parental Abs to gp140 and gp160, which express both epitopes, suggesting that bispecific binding may have occurred.

To test binding to a more physiologically relevant antigen, we used indirect immunofluorescence and flow cytometry to analyze binding of DVD-Igs to persistently infected H9/NL4-3 cells (figure 2B and 2C). Addition of sCD4 enhances binding of anti-gp41 mAbs, but diminishes binding of anti-gp120 [13], [14], and this is shown in figure 2B. By analyzing binding in the presence or absence of CD4, we may infer which Env domain is most responsible for DVD-Ig binding. All 7B2-[Fx]-HY DVD-Igs behaved like the 7B2 parent, i.e. binding was enhanced by sCD4. But no DVD-Ig exceeded the binding of either parental Ab. Since ELISA results indicated potential binding activity of the inner domain, we wished to further test the ability of each binding site to function independently and simultaneously. Flow cytometry was used to measure competitive inhibition of binding to infected cells by gp41 peptide or rgp120 (figure 2D). Increasing concentrations of Abs were added to 10 µg/ml of a single Ag or a mixture of 10 µg/ml of each Ag, and then incubated with H9/NL4-3 cells. The soluble antigen completely inhibited the binding of the parental Abs to the cells. 7B2-[Fx6]-HY could bind to cells in the presence of either inhibitor, indicating that one domain could be fully occupied, and the other still be capable of binding antigen. However when gp41 was blocked, only minimal binding to cells was observed, suggesting that the outer domain encoded by 7B2 dominated binding activity. In accordance with ELISA results, the shorter 7B2-[Fx2]-HY bound only gp41.

**Figure 3:**
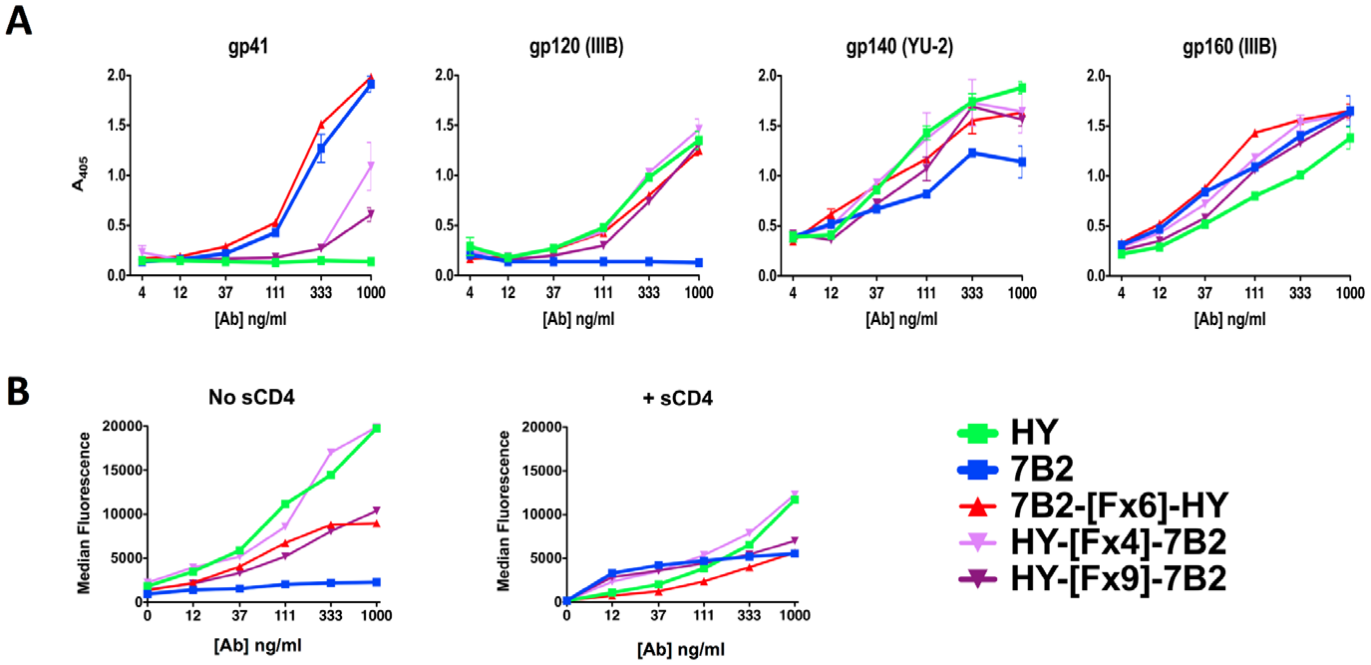
Effect of domain orientation on the binding of DVD-Igs to Env. ELISA (panel A) and flow cytometry (B) were used to evaluate the effects of domain orientation. Binding of DVD-Igs with 7B2 as the external domain was compared to DVD-Igs with HY as the external domain. Experiments were conducted as described in Figure 2. ELISA results are representative of at least 3 assays, FACS of 2.

To determine whether altering the domain orientation would influence the binding characteristics of the DVD-Igs, we tested constructs expressing HY as the outer domain linked by 4 or 9 repeats of [GGGGS]. Binding profiles of HY-external DVD-Igs by both ELISA and FACS resembled those of the HY parent (figure 3). Of note, HY-[Fx9]-7B2 with 9 repeats of flexible linkers demonstrated weaker binding than HY-[Fx4]-7B2, suggesting that too large a linker may be a hindrance.

**Figure 4:**
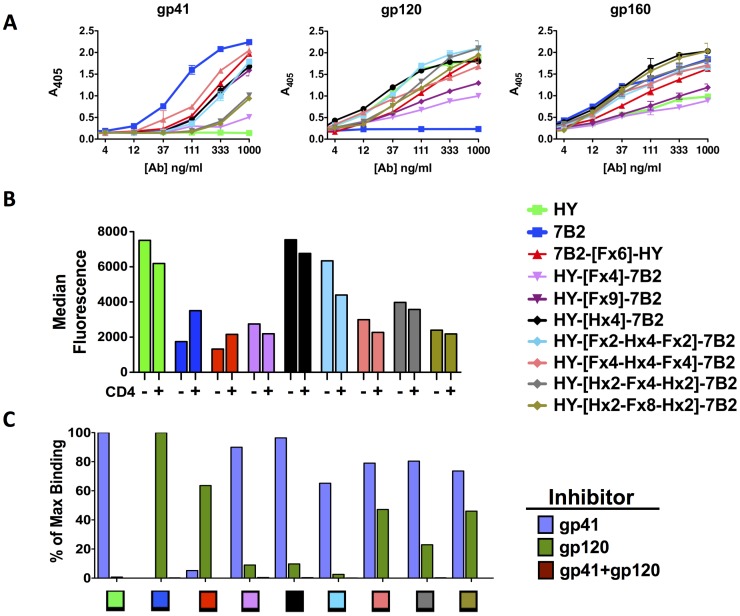
Effect of linker type on binding to Env. ELISA (A) and flow cytometry (B and C) were used to evaluate the binding of DVD-Igs with different linker types, linker lengths, and domain orientations to Env. Abs are identified by the same color in each panel. Methods are as in Figure 2. *A.* ELISA binding to protein/peptide Ag. *B.* Binding of Abs to persistently-infected H9/NL4-3 cells was detected by flow cytometry. Median fluorescence is reported for Abs tested at 10 µg/ml in the presence or absence of sCD4 (300 ng/ml) as noted along the x-axis. *C.* Inhibition of Ab binding to H9/NL4-3 cells by soluble gp41 and gp120 Ags. Flow cytometry results are shown as percent of maximal binding (binding in absence of inhibitor). Ags were used at 10 µg/ml, Abs at 0.6 µg/ml. Binding in the presence of both Ags was less than 1%. ELISA and FACS studies are representative of at least 4 separate assays.

We next tested the effect of including helical domains in the linker. Helical domains have rigidity and thus add stability and defined spacing. One DVD-Ig had a helix-only linker (Hx4). Others had linkers with combinations of flexible and helical sequences. All helix-containing constructs were made with the gp120 binding HY as the external domain. Figure 4 compares the helix-containing DVD-Igs to the best flexible linker constructs. We first studied the effects of linker type on ELISA binding (figure 4A). Helical linkers could not completely overcome the hindrance of the inner domain’s binding to gp41, although they were clearly better than the HY-external constructs with flexible linkers only. Helix-containing DVD-Igs bound gp120 as well as the HY parent and showed enhanced binding to gp160, presumably through a bispecific interaction. Flow cytometry was performed on H9/NL4-3 cells to compare the binding of Abs in the presence and absence of sCD4 (figure 4B). The outer domain of the helix-containing DVD-Igs defined their binding characteristics, i.e. better binding was observed in the absence of sCD4, than in its presence. Two of the helical constructs, HY-[Hx4]-7B2 and HY-[Fx2-Hx4-Fx2]-7B2, outperformed the other DVD-Igs. To determine if both domains can bind antigen at the same time, we used soluble Ag to inhibit the binding of DVD-Igs to cells (figure 4C). HY and 7B2 were completely blocked by the appropriate inhibitor, but not the irrelevant one, whereas the helical domain DVD-Igs bound to infected cells in the presence of one inhibitor, but were completely blocked in the presence of both. These results demonstrate that both domains can bind both cognate antigens simultaneously.

**Figure 5:**
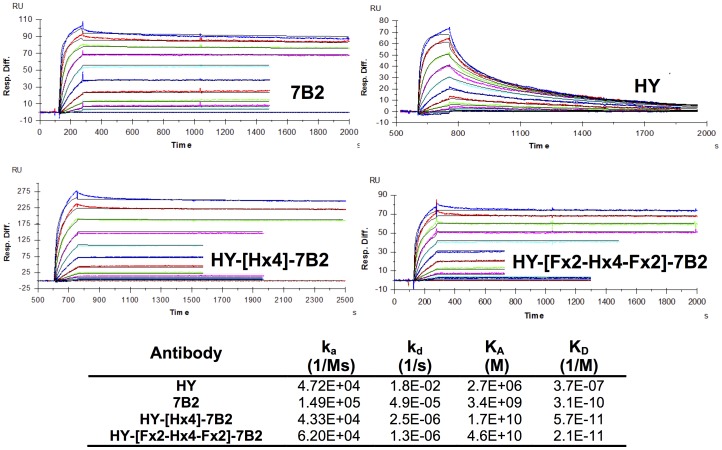
Measurement of binding of DVD-Igs to rgp160 by surface plasmon resonance. Recombinant gp160 was immobilized on sensor chips. Two-fold dilutions of each Ab (400 to 0.39 nM) were run on the chips serially, and Ab desorbed between runs. Ab flowed over the chips for 2.5 min, followed by buffer for 20 min (HY and low concentrations of 7B2 and DVD-Igs) or 60 min (high concentrations of 7B2 and DVD-Igs). The latter parts of the 60 min curves are not shown. Protein bound to the chips (in response units) is shown on the vertical axis, time (sec) on the horizontal. The curves shown are representative of 3–6 replicate runs. Curves fit best to a bivalent analyte binding model for calculation of rate constants.

Avidity of DVD-Ig binding to recombinant gp160 was measured in real time using surface plasmon resonance (figure 5). Because HY Ab binds to an epitope that is conformationally sensitive, and it is unlikely that the recombinant gp160 used in this assay retains the native conformation, we believe this results in an artificially lower binding constant, due to the more rapid dissociation rate. However, even with this, it is clear that K_D_ of the DVD-Igs is improved by an order of magnitude over the binding of the parental Abs. If the outer domain is the dominant binding domain, as the ELISA and FACS results indicate (figure 4), then the addition of the inner 7B2 domain to these DVD-Igs results in a marked improvement in overall function of the Ab over that of HY alone.

### Immunoconjugate Cytotoxicity and Virus Neutralization

**Figure 6:**
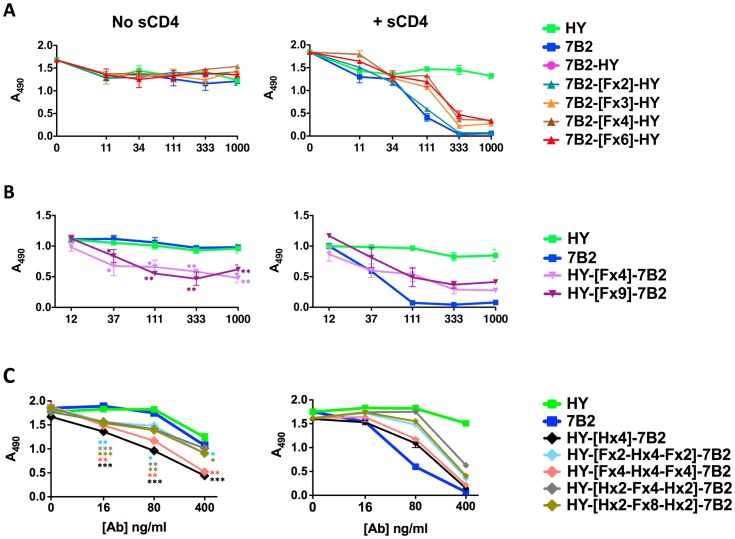
Delivery of a cytotoxic immunoconjugate by DVD-Igs. The ability of DVD-Igs to target and kill infected cells was examined using an indirect immunoconjugate (IC) killing assay. H9/NL4-3 cells were incubated for 1 hr with Abs in the presence (R) or absence (L) of sCD4 (300 ng/ml), followed by addition of 500 ng/ml ricin A chain-conjugated anti-IgG secondary Ab. After 3 days, cell viability was measured at 490 nm after adding MTS/PMS. Data are plotted as absorbance versus Ab concentration. *A.* DVD-Igs with 7B2 as the outer domain, using flexible linkers. *B,* DVD-Igs with HY as the external domain and flexible linkers. *C.* DVD-Igs with HY as external domain and helical linkers. Each DVD-Ig was tested in 2–4 separate experiments, shown here is one representative experiment for each set of DVD-Igs. Statistically significant improvements in DVD-IC activity, when compared to the effective parental Ab 7B2, are indicated by asterisks (*p<.05, **p<.01, ***p<.001, by one-tailed t test).

We have studied the biological activity of the DVD-Igs in two ways: neutralization of virus infectivity and the ability to deliver cytotoxic conjugates to HIV-infected cells. To test cytotoxic activity, we use an indirect immunoconjugate assay [54], [55]. The H9/NL4-3 cells used in the assays maintain nearly 100% infection in cell culture (see figure 2B). Infected cells were first incubated with differing concentrations of Ab, then a toxin-conjugated anti-IgG secondary Ab was added. Cell viability was measured after 3 days by MTS dye reduction, with a decrease in A_490_ indicating cell death (figure 6). As in binding assays, DVD-Igs functioned most like the parent donating the outer domain. In the case of 7B2-[Fx]-HY, all constructs regardless of linker length were cytotoxic to infected cells in the presence of sCD4, but remained ineffective in the absence of sCD4, analogous to 7B2 itself (figure 6A). However, when domains were swapped in the HY-[Fx]-7B2 DVD-Igs, DVD-Igs were cytotoxic (40–60% killing) with or without sCD4, indicating the arrangement of the additional domain added to the cytotoxic activity not present in the other orientation (figure 6B). We next tested the DVD-Igs with helical linkers (figure 6C). In the absence of sCD4, all helix-containing DVD-Igs outperformed both parents. In the presence of sCD4, all of the helical DVD-Igs outperformed the HY parent Ab, and some approached the activity of 7B2.The ability of the HY-external DVD-Igs to target ICs in the absence of CD4 represents a statistically significant improvement in the function that is the primary aim of these studies.

**Figure 7:**
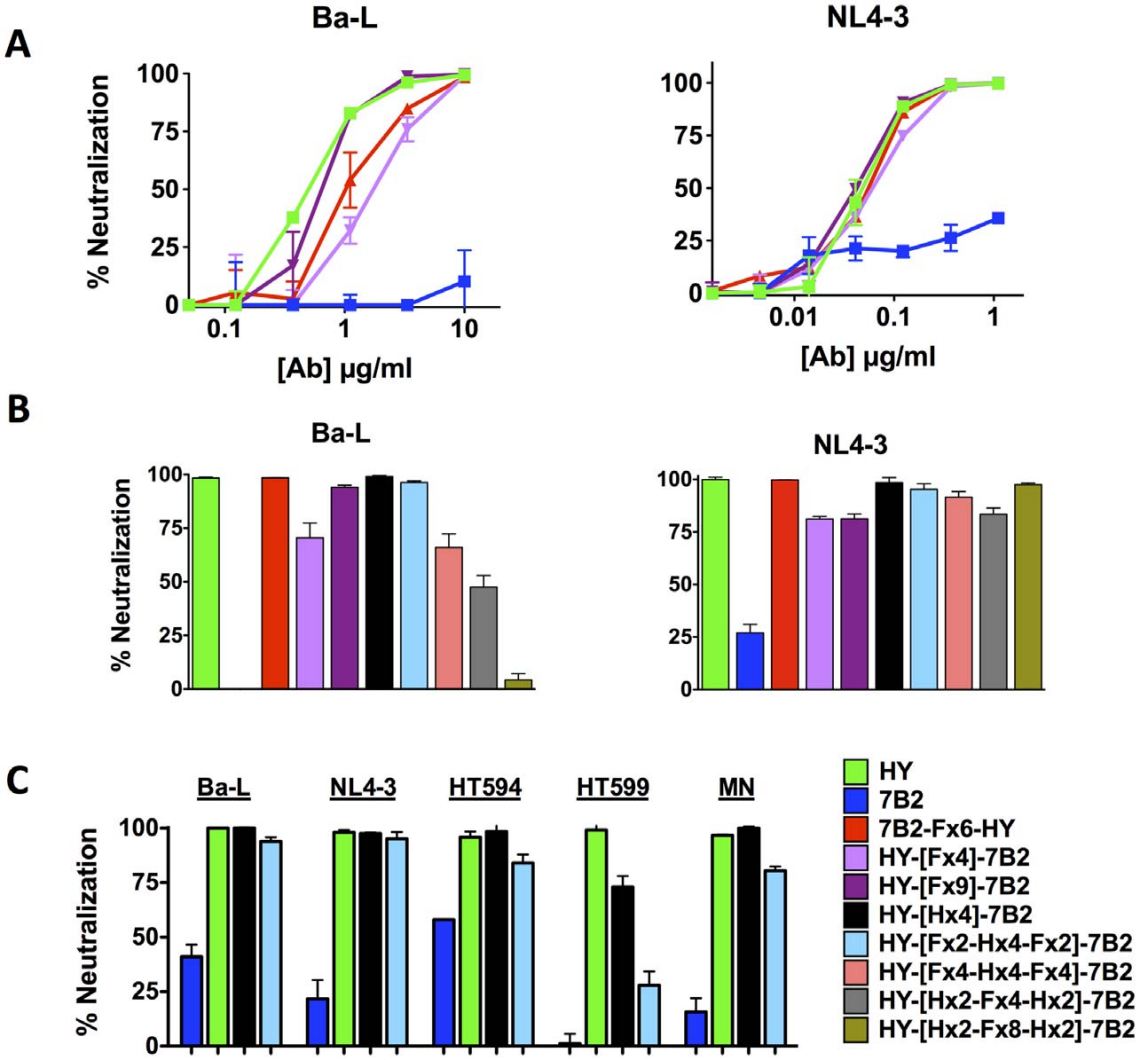
Neutralization of HIV infectivity by DVD-Igs. The ability of the DVD-Igs to neutralize different isolates of HIV was tested using the TZM-bl assay. *A.* Antibodies were titrated. *B. and C.* Antibodies were tested at 10 µg/ml, except against NL4-3 which was tested with 1 µg/ml. Ab and a predetermined dilution of virus were mixed and transferred to a monolayer of TZM-bl cells. Three days later, infectivity was read as chemiluminescence. Results are displayed as percent neutralization (virus/no Ab = 0%; no virus = 100% neutralization) according to the formula: [1-(RLU_Ab_-RLU_bkgrd_)/(RLU_NoAb_-RLU_bkgrd_)]*100.

We also tested the ability of the DVD-Igs to neutralize HIV infectivity, even though only one of the parental Abs (HY) is a “neutralizing” Ab. We tested both X4 and R5-tropic HIV isolates using a TZM-bl luciferase assay. Viruses were pre-mixed with dilutions of antibody, then incubated with TZM-bl cells for 3 days, and luciferase was assayed (figure 7). Of the parental Abs, HY is known to be a broadly neutralizing antibody [60], [61], whereas 7B2 has marginal, if any, ability to neutralize HIV, and our results in figure 7 are consistent with these observations. 7B2-[Fx]-HY constructs with linkers shorter than 4 repeats did not neutralize (data not shown). In figure 7A, the DVD-Igs with flexible linkers were titrated against two commonly used HIV laboratory isolates: NL4-3 and Ba-L. NL4-3 has been characterized as highly sensitive to neutralization, and this is reflected in the concentration of Ab necessary to neutralize NL4-3, compared to that needed for Ba-L and the other isolates. 7B2-[Fx6]-HY and both HY-[Fx]-7B2 DVD-Igs showed neutralization activity similar to HY. In figure 7B we test a broad panel of Abs for neutralization of the NL4-3 and Ba-L isolates. Of the DVD-Igs with helical linkers, the two that exhibited the best binding and cytotoxic activity, HY-[Hx4]-7B2, and HY-[Fx2-Hx4-Fx2]-7B2, also demonstrated the best neutralization (figure 7B). These two DVD-Igs were then tested against a wider panel of HIV isolates (figure 7C). Although neutralization activity was observed, these antibodies were not as efficient as HY itself. Thus, there was no evidence of enhanced neutralization by the addition of the 7B2 domain, which is not surprising, given that 7B2 has little neutralization activity of its own. It is also possible that the addition of 7B2 domain was a hindrance to neutralization, as seen best against the isolate 92HT599. However our goal was not necessarily to produce Abs with better neutralization ability, but rather to make effective ICs, and that guided our choice to use the 7B2 V-region in the DVD-Igs.

## Discussion

We are developing immunoconjugates (ICs) to be used for the eradication of HIV infection in patients using activate-and-purge protocols, where latently infected cells are first activated to express HIV, and then eliminated [16]–[22]. Although viral cytopathic effect could result in elimination of activated cells, we believe that the targeted cytotoxicity of ICs will increase the efficiency of purging infected cells. Env protein, the only viral protein expressed on the surface of infected cells, would be the target of such ICs. We have examined over 100 anti-Env mabs for efficacy in targeting ICs, and have identified a key epitope on gp41, defined by mAb 7B2, as the best target [10]–[15]. Abs to other epitopes on Env, including the CD4 binding site and V3 loop, as well as CD4 itself, have also been used to target ICs [10], [24], [25], but are less effective when directly compared to 7B2 in the presence of sCD4, see figure 6, and references [12], [14], [15]. Over the past several years, a host of potent broadly neutralizing Abs to HIV gp120 have been developed [62]–[65], but in side-by-side assays as ICs, none is as effective as 7B2+ sCD4 (S.H. Pincus, unpublished data). To determine if IC efficacy may be improved by Ab engineering approaches, we have designed DVD-Igs that target both gp41 and the CD4 binding site. We show that bifunctional binding of both gp120 and gp41 can be obtained and that IC targeting is improved, particularly in the absence of sCD4. There is no improvement in the ability of the DVD-Igs to neutralize HIV when compared to the HY parent, however all are markedly better than the poorly neutralizing parent 7B2. Interestingly even though the outer domains dominate binding activity in most aspects, neutralization by 7B2-[Fx6]-HY more closely resembles the neutralization of the HY parent rather than 7B2, which donated the outer domain.

We have examined the effects of linker length and design, and the orientation of V domains on the properties of the DVD-Igs. The [GGGGS]n linker [Fx] was chosen due to its flexibility and proven effectiveness [33], [52], [57]–[59]. We also incorporated repeats of an [AEEEK]_n_ helical linker [Hx] alone or in combination with the flexible linkers. The helical sequence is noteworthy for its stability and discrete spacing ability [33], [52], [57]. In our studies, shorter linkers (<15 amino acids) were insufficient to allow the inner domain to function, while longer linkers allowed greater access for the inner domains to bind antigen. Because the parental Abs have marked differences in binding to cell-surface Env, neutralization, and the ability to deliver ICs, we were able to evaluate the contribution of each domain to the function of the DVD-Ig. To do this, we constructed DVD-Igs with either 7B2 or HY as the external V-domain. As we have noted, the external domain is dominant in defining the function of the DVD-Igs. However clear-cut contributions of the inner domain were demonstrated. The addition of 7B2 variable domain markedly enhanced the ability of the HY-external DVD-Igs to deliver ICs when compared to the parent HY Ab (figure 6). Similarly, the HY internal domain, markedly improves the neutralization ability of 7B2-[F×6]-HY when compared to 7B2 alone (figure 7). Adding the second variable domain improves binding avidity (figure 5). Bifunctional binding by DVD-Igs may also hinder the function of Env by cross-linking gp120 and gp41, as postulated by Mouquet, et al [39].

**Figure 8:**
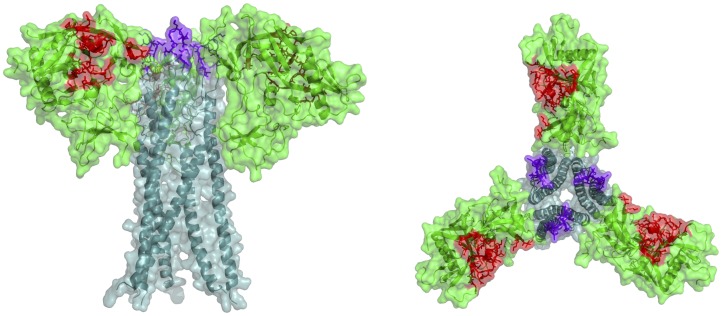
Model of Env showing Ab binding sites. Gp120 is shown in green with the variable loops deleted, gp41 in blue, the HY epitope in red, and 7B2 in violet. Molecular modeling was performed starting with the crystal structures of gp120 [67], [68] and homology model of HIV gp41. The sequence of HIV gp41 clade B was built onto the homologous NMR structure of SIV gp41 [69] using Modeller [70], allowing the structure of the loop region of HIV gp41, and thus the location of the 7B2 epitope, to be approximated. The relative position of the gp120 trimer to the gp41 trimer was constructed by searching through the rotational and translational degrees of freedom about the threefold symmetry axis. The structure shown is the complex with the lowest potential energy using the Amber03 Forcefield [71]. A thorough description of the modeling protocols and energetic analyses will be published elsewhere (M.A. Zubieta and C.M. Summa, manuscript in preparation).

We have shown concurrent binding of the DVD-Igs to both gp120 and gp41. ELISA binding of the best DVD-Igs to gp140 and gp160 (which express both epitopes) exceeded that of the parental Abs, whereas binding to either gp120 or gp41 did not (figure 4A). Similarly, only partial inhibition of DVD-Ig binding to cell-surface Env was obtained with concentrations of soluble gp120 or gp41 that completely inhibited the binding of the parental mAb, whereas a mixture of both gp120 and gp41 completely inhibited (figures 2D and 4C). Because the two target epitopes are on distinctly different domains of Env, it was not *a priori* obvious that bifunctional binding would be obtained on native Env, although the work of Mouquet, et al provides precedent [39]. There is no atomic level for the intact gp120/gp41 trimeric structure. The loop region of gp41, where 7B2 binds, is not represented on any crystal or NMR structure of the molecule, even though it is the immunodominant domain of gp41. We have used molecular modeling to generate a hypothetical 3-D structure of the native Env trimer including the gp41 loop, and to map the epitopes of HY and 7B2 onto the structure (figure 8). The results show that these epitopes are sufficiently proximate to allow binding by both V domains, if there is adequate linker length. As shown in figure 2D, a linker of two flexible domains is insufficient to allow both domains to bind concurrently, whereas six domains allow both domains to do so.

Despite all of our modifications, we have not observed clear evidence of additive or synergistic effects of bispecific binding on the function of the DVD-Igs. One potential explanation is that the DVD-Ig format does not allow sufficient flexibility for optimal binding to both epitopes. The differences we have observed by altering the inter-V-domain linkers support this idea. Thus it is possible that by altering the form of the bifunctional Ab, enhanced functional activity may occur. Mouquet, et al. [39] have produced a conventional bifunctional Ab, using knob-and-hole technology, and Fvs targeting Env epitopes similar to those used here. They have reported enhanced neutralization activity, compared to the parental Abs alone. These data suggest that the bifunctional Ab format may serve better for simultaneous targeting of the CD4 binding site and the gp41 external loop region. Because Mouquet used different parental Abs than reported here, it is not possible for us to perform a direct comparison of these two formats using existing reagents.

DVD-Igs are large molecules. Studies have shown that the addition of a second set of V domains does not alter in vivo pharmacokinetics [34]. In oncology, where tumor penetration is often an important concern, the drive has been to design smaller ICs, based on scFv or even smaller single domain molecules. However, when targeting cells of the lymphoid system, penetration is not as much of a concern [66]. We have observed that the in vivo efficacy of anti-HIV ICs is enhanced by the use of intact Ig, rather than Fab or scFv (reference [15], and S.H. Pincus, unpublished), presumably a result of increased plasma residence. Although it should be noted that intact Ab HY does not function well as an immunoconjugate (figure 6), while an scFv IC utilizing the same V-regions is highly effective [21], [23], suggesting that access to the target of the HY Ab may be hindered by size of the targeting molecule.

In summary, we have produced a series of DVD-Igs with variable domains that bind to both gp120 and gp41. We have explored the effects of altering the inter-V-domain linker, as well as the orientation of the two V domains, on the ability of the DVD-Igs to bind to recombinant antigens and HIV Env expressed on the surface of infected cells, to neutralize infectious HIV, and to deliver cytotoxic immunoconjugates. Cell binding and immunoconjugate testing were examined both in the presence and absence of sCD4. The two parental Abs differ markedly in these functions. We have found that the DVD-Igs generally perform each of these functions as well as the more effective parent, and outperform the less effective. We have identified DVD-Igs that deliver cytotoxic ICs more effectively than either parent, our primary aim in designing these novel antibodies.
